# Using principal component analysis to reduce complex datasets produced by robotic technology in healthy participants

**DOI:** 10.1186/s12984-018-0416-5

**Published:** 2018-07-31

**Authors:** Michael D. Wood, Leif E. R. Simmatis, J. Gordon Boyd, Stephen H. Scott, Jill A. Jacobson

**Affiliations:** 1 Centre for Neuroscience Studies, Queen’s University, Botterell Hall, 18 Stuart St, Kingston, ON Canada; 2 Department of Critical Care Medicine, Queen’s University, Kingston, ON Canada; 3 Department of Medicine, Queen’s University, Kingston, ON Canada; 4 Department of Psychology, Queen’s University, 318 Craine Hall, 62 Arch Street, Kingston, ON K7L 3N6 Canada

**Keywords:** KINARM, End-point robot, Principal component analysis, Data reduction

## Abstract

**Background:**

The KINARM robot produces a granular dataset of participant performance metrics associated with proprioceptive, motor, visuospatial, and executive function. This comprehensive battery includes several behavioral tasks that each generate 9 to 20 metrics of performance. Therefore, the entire battery of tasks generates well over 100 metrics per participant, which can make clinical interpretation challenging. Therefore, we sought to reduce these multivariate data by applying principal component analysis (PCA) to increase interpretability while minimizing information loss.

**Methods:**

Healthy right-hand dominant participants were assessed using a bilateral KINARM end-point robot. Subjects (*N*s = 101–208) were assessed using 6 behavioral tasks and automated software generated 9 to 20 metrics related to the spatial and temporal aspects of subject performance. Data from these metrics were converted to Z-scores prior to PCA. The number of components was determined from scree plots and parallel analysis, with interpretability considered as a qualitative criterion. Rotation type (orthogonal vs oblique) was decided on a per task basis.

**Results:**

The KINARM performance data, per task, was substantially reduced (range 67–79%), while still accounting for a large amount of variance (range 70–82%). The number of KINARM parameters reduced to 3 components for 5 out of 6 tasks and to 5 components for the sixth task. Many components were comprised of KINARM parameters with high loadings and only some cross loadings were observed, which demonstrates a strong separation of components.

**Conclusions:**

Complex participant data produced by the KINARM robot can be reduced into a small number of interpretable components by using PCA. Future applications of PCA may offer potential insight into specific patterns of sensorimotor impairment among patient populations.

**Electronic supplementary material:**

The online version of this article (10.1186/s12984-018-0416-5) contains supplementary material, which is available to authorized users.

## Background

Robotic technology, such as KINARM (BKIN Technologies, Kingston, ON, Canada), provides objective metrics using the participants’ upper limbs to assess proprioception and sensorimotor function, as well as executive function. In stroke survivors, this technology has identified subtle neurocognitive deficits not apparent on routine clinical testing [1], and various KINARM tasks have been administered to multiple patient populations (e.g., traumatic brain injury, fetal alcohol spectrum disorder) [2–5]. The KINARM behavioral battery (KINARM Standard Tests™) currently consists of 9 tasks that include automated data analysis routines. Within each task, up to 20 performance items (e.g., reaction time) are calculated, leading to the potential generation of over 100 metrics per participant. However, when assessing performance deficits that are indicative of adverse neurological outcomes among participants, this granular and complex performance output may impede deriving meaningful interpretations.

Principal component analysis (PCA) is a data reduction technique used to identify linear combinations of measured variables that account for the most overall variance in responses [6–8]. The first principal component accounts for the largest amount of variance, followed by the second, and so forth [9]. PCA is best used when the measured variables are theorized to be causal or formative indicators of the overarching construct rather than reflective or effects of it, which would be better assessed using factor analysis (FA). Because performance on the tasks determine a participant’s level of functioning rather than the other way around, PCA is the more appropriate technique for the KINARM battery than is FA.

The primary objective of this study was to reduce the dimensionality of healthy participant data produced by the KINARM End-Point robot by using PCA. This analysis should increase overall interpretability by reducing redundant KINARM parameters into behaviorally meaningful components, which has the potential to demonstrate the clinical utility of PCA by capturing a minimal number of performance measures that could assist with the characterization of deficits among various patient populations.

## Methods

### Participant recruitment

Healthy participants were community-based and were randomly recruited via advertisements on lab and departmental websites, as well as in local classifieds (online and print). Trained research staff screened each adult participant (> 17 years old) to ensure that task instructions could be easily understood, no prior neurological deficits were reported, and that subjects had no prior medical conditions that could affect upper limb mobility (see Additional file 1). Once enrolled, participants were then assessed by our research staff at one of two sites: Kingston Health Sciences Centre or Queen’s University, both sites in Kingston, ON, Canada. See Table 2 for detailed demographics (e.g., age, gender, education). The Queen’s University and Affiliated Hospitals Health Sciences Research Ethics Board approved recruitment and assessment of these participants. Informed consent was obtained from each participant prior to the KINARM assessment.

### Robotic assessment

**Fig. 1:**
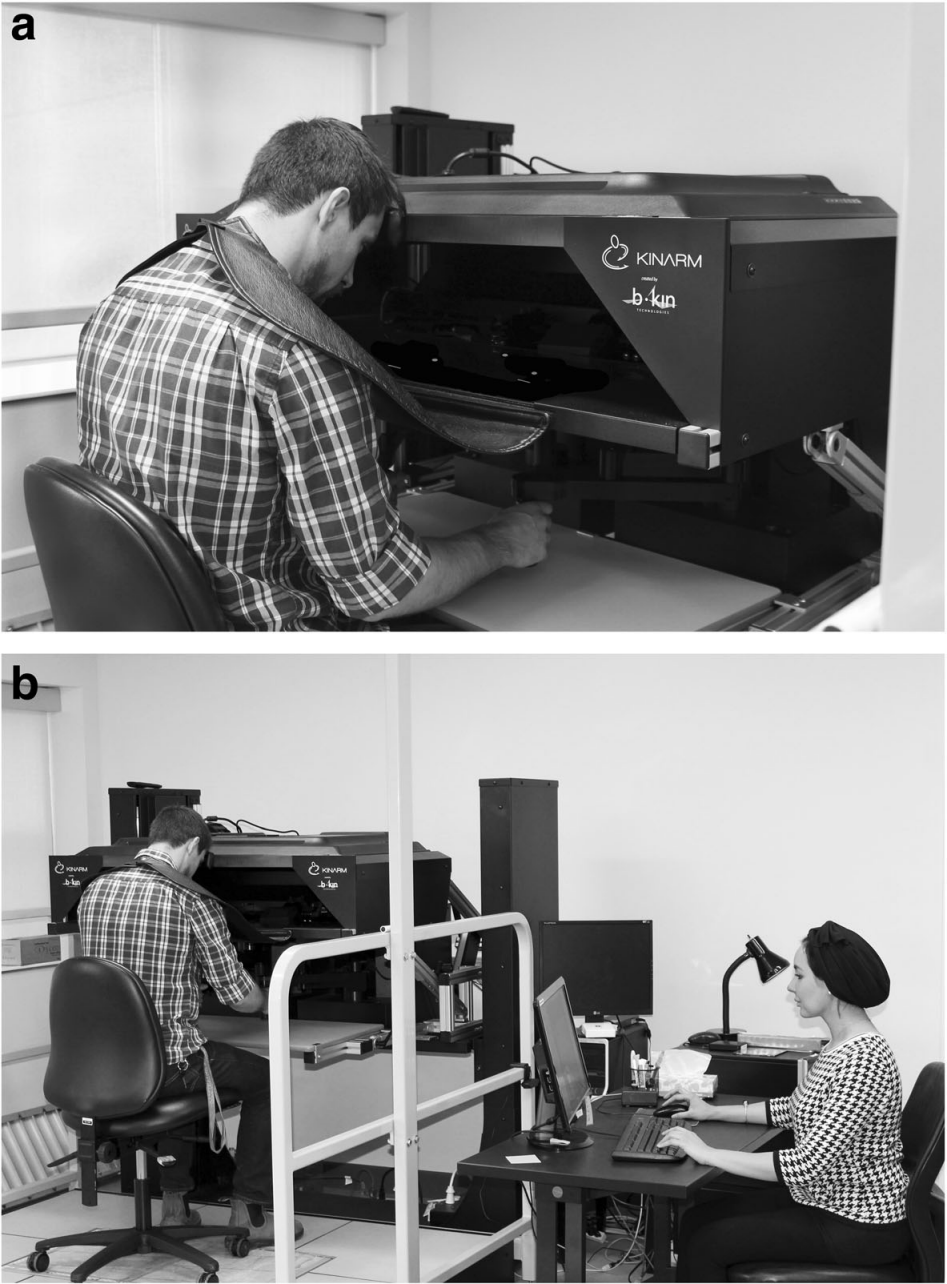
KINARM End-Point robot set-up during participant assessment

Participants were seated, in a height-adjustable chair that was locked in place, at a two-dimensional virtual reality system that displayed each task in the horizontal plane. As shown in Fig. 1, participants’ vision of their hands and arms was occluded, their head was positioned in the center of the visual field, and visual feedback of hand position (when provided) was represented on the screen by a white circle. Participants were instructed to grasp onto the KINARM End-Point robotic handles (BKIN Technologies Ltd., Kingston, ON, Canada), permitting free movement in the horizontal plane without anti-gravity support at proximal or distal arm segments. A trained operator described each task, using a standardized script, before it was performed by the participant. Automated data collection and analysis software (Dexterit Version 3.6.2) measured and quantified subject performance. For each performance metric, the software computed a Z-score that accounted for age, sex, and handedness. Only performance metrics that could be normalized, and therefore converted to Z scores, were considered in the current analysis. We examined 6 tasks from the KINARM Standard Tests™, four tasks assessed upper-limb motor function in right-handed participants: visually guided reaching (VGR), object hit (OH), object hit and avoid (OHA), and level 1 of ball on bar (BonB). The Arm position matching (APM) task assessed upper limb proprioception, and reverse visually guided reaching (RVGR) assessed cognitive-motor function. For detailed descriptions of all tasks, see Table 1**.** Within each task, approximately 9–20 performance metrics were produced, participants were instructed to take breaks as needed, and the assessment took < 1 h to complete. For detailed descriptions on all task parameters, see Additional file 2 or obtain from the BKIN Technologies Ltd. KINARM manual [10]. All included participants were selected based on their ability and understanding to follow task commands. Each task was comprised of various healthy participants, as not all participants completed each task. To reduce handedness as a potential confounding variable, only right hand dominant subjects were included in the analysis.

**Table 1:**
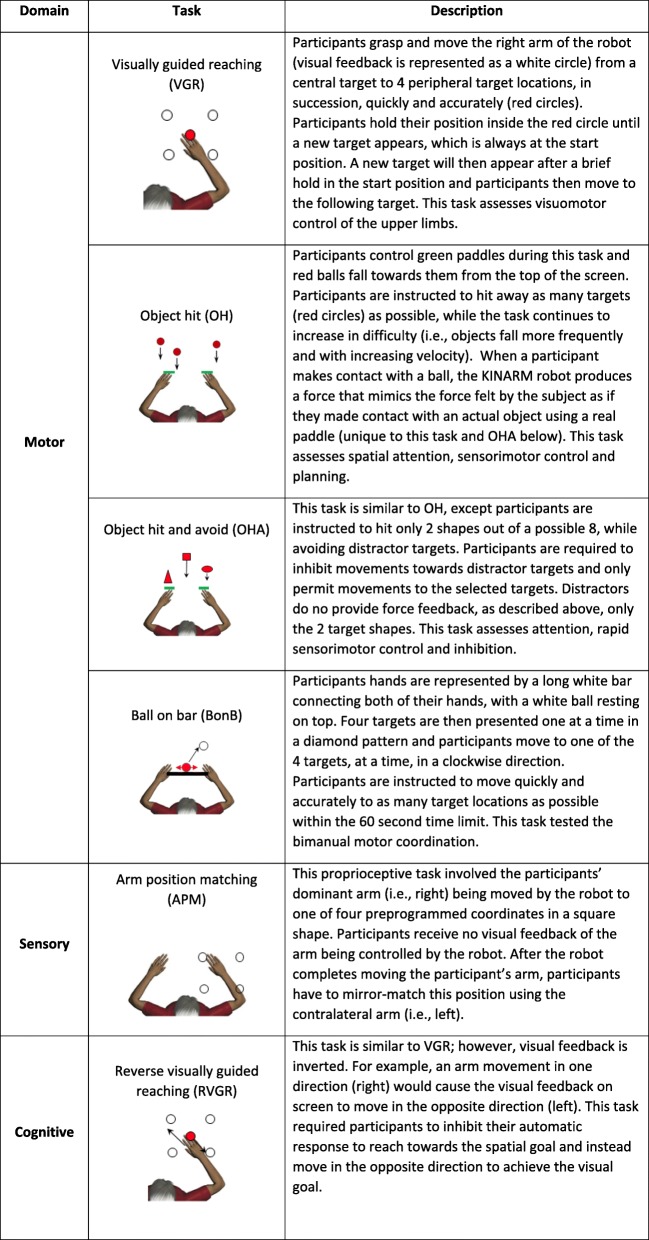
KINARM Task Descriptions and Domains of Assessment

### Principal component analysis

PCA analysis was conducted in R version 3.3.2 [11] using the psych package version 1.7.8 [12]. KINARM performance items were converted to Z-scores prior to PCA. For correlation matrices associated with each task, see Additional file 3. Scree plots and parallel analysis were examined to determine the number of components per KINARM task. Briefly, eigenvalues (variance explained by a component) were plotted in descending order to generate a scree plot [13, 14] per KINARM task. Parallel analysis was used to generate a random data set that possessed the same number of items and sample size as each KINARM task. Eigenvalues then were calculated for the random data, which also are graphed on the scree plot mentioned previously. The number of components prior to where the random data crosses the observed KINARM participant data are retained (i.e., retain the number of components that have eigenvalues larger than those from the simulated dataset) [13–16]. In PCA, if more than one component is retained, then multiple solutions for the parameters would fit the data equally well. Rotation is used to help select the best solution. An oblique rotation (oblimin) [17], which allows for components to be correlated, first was conducted for each solution. If all intercomponent correlations were low (<|0.30|), then an orthogonal rotation (varimax) [18] was used instead. A component loading was considered substantial if the loading was ≥0.40. Only participants who were right hand dominant were chosen for this analysis, and only data from their dominant hand were used for PCA. To reduce possible practice effects for those participants who completed multiple assessments, we used the participant performance metrics from the *first* KINARM assessment only (i.e., for participants who completed the same task multiple times [repeated testing], we used their performance score only from the first assessment of that task). These identified components were then analyzed for interpretability, which was defined as parameters having substantial loadings on each component and groupings of performance parameters having behaviorally meaningful conclusions (e.g., the constructs of “*total movement*” and “*posture/reaction time*” being separable). To further characterize participant performance, components and their respective parameters that loaded highly were used to broadly generate intuitive labels for each component per task rather than successively numbering components.

## Results

### Participant characteristics

The number of participants across each task varied (Ns = 101–208). Participant level of education consistently ranged from high school education to postdoctoral researcher. The percent of participants that were male ranged from 40 to 46%, and the mean age of subjects (range: 41–46 years) was fairly consistent across all tasks. Only a small subset of participants (*n* = 11) completed all 6 KINARM tasks. For more detailed demographics per task, see Table 2.

**Table 2:** Participant Demographics and Task Characteristics

Task	N	Mean Age (range)	Male Gender (%)	Dominant Arm	Range of Education	Mean Number of Trials [SD]	Mean Task Duration in seconds [SD]
Arm Position Matching	184	44.43 (18–87)	84 (46)	Right	High school – Postdoctoral research**	25.26 [0.98]	88.13 [19.41]
Ball on Bar	208	42.16 (18–87)	84 (40)	Right	High school – Postdoctoral research***	1	62.57 [1.60]
Visually Guided Reaching	200	42.51 (18–88)	80 (40)	Right	High school – Postdoctoral research***	24 [0.07]	102.55 [6.83]
Reverse Visually Guided Reaching	101	41.56 (19–81)	46 (46)	Right	High school – Post-doctoral research	30 [0.10]	180.27 [16.71]
Object Hit	190	46.28 (18–87)	86 (45)	Right	High school – Postdoctoral research**	1	138.03 [0.43]
Object Hit and Avoid	170	45.56 (18–87)	76 (45)	Right	High school – Postdoctoral research*	1	138.29 [0.51]

For range of education, High school reflects completion of grade twelve or completion of secondary school equivalent. * indicate the number of participants that were missing data for that column. For example, *(*n* = 1), **(*n* = 2), *** (*n* = 3)

### Data reduction

The number of KINARM performance parameters, per task, were substantially reduced (range 67–79%) by grouping related parameters into components. APM, BonB, VGR, RVGR, and OH reduced from 9 to 14 performance metrics to 3 components, which accounted for 70–79% of variance. OHA was reduced from 20 performance items to 5 components, while still accounting for 82% of the variance (Table 3).

**Table 3:**
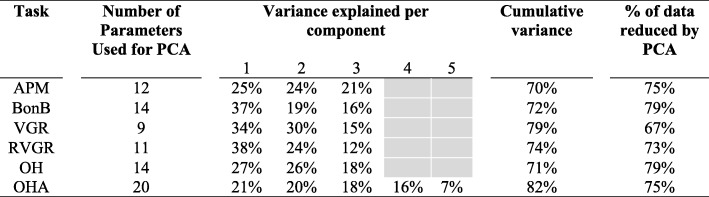
Proportion of variance explained by each component across KINARM tasks

Grey shaded region indicates that there are no components for these tasks. *APM* Arm position matching, *BonB* Ball on bar, *VGR* Visually guided reaching, *RVGR* Reverse visually guided reaching, *OH* Object hit, *OHA* Object hit and avoid

### Principal component analysis across the KINARM battery

**Fig. 2:**
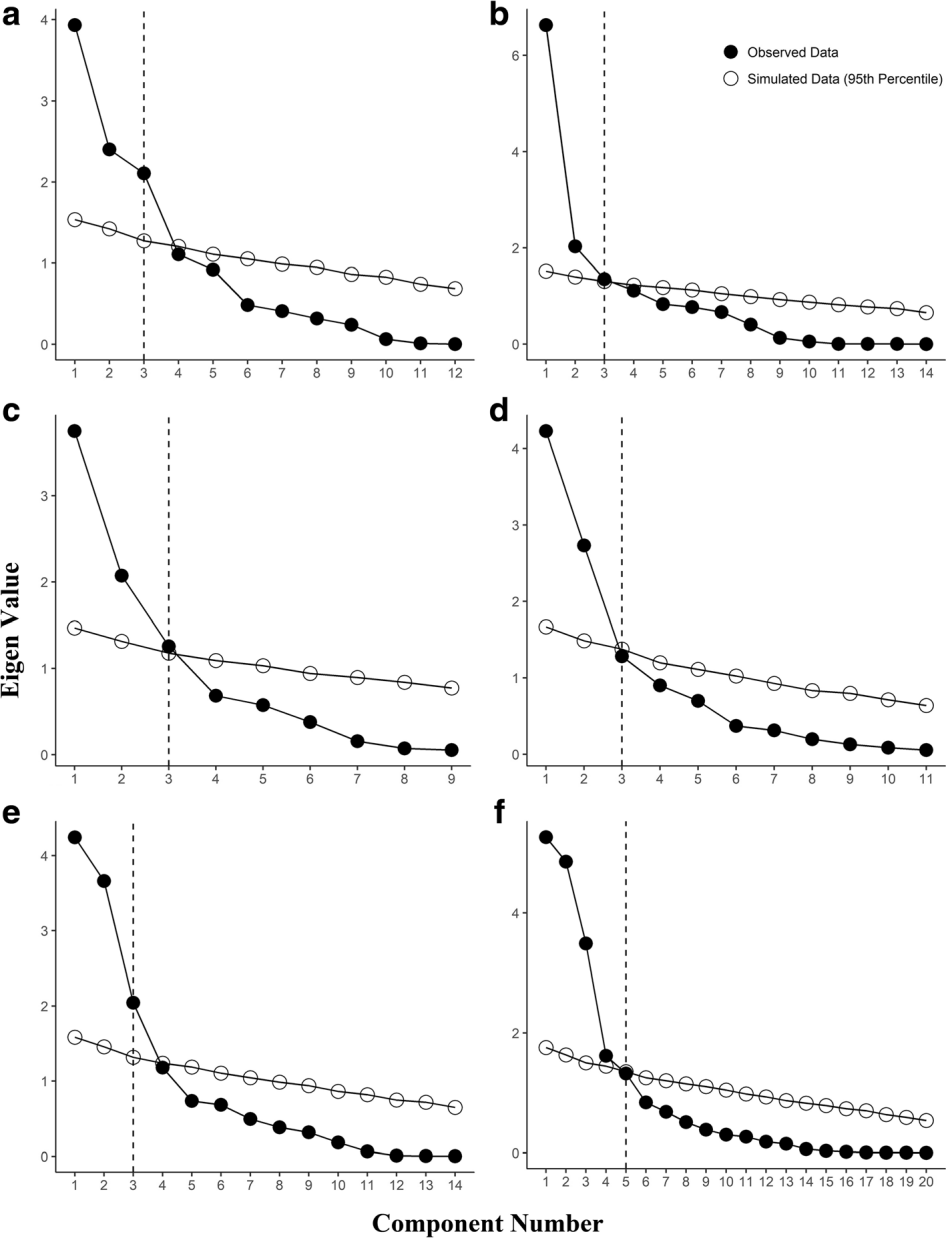
Scree plots with parallel analysis indicating the number of components to be selected across KINARM tasks. **a** Arm Position Matching; **b** Ball on Bar; **c** Visually Guided Reaching; **d** Reverse Visually Guided Reaching; **e** Object Hit; **f** Object Hit and Avoid

Descriptions of each task and their respective components are provided below as follows: component name (number of KINARM parameters contributing to that component [according to the ≥.40 load criteria mentioned previously], percent of variance explained by that component). See Table 4 for component loadings per task, all available items (i.e., metrics that could be converted to Z scores) were considered for each PCA per task. For scree plots with parallel analysis per task, see Fig. 2.

**Table 4:** Principal component loadings for KINARM behavioral battery

Task	KINARM Items	Component Loadings				
		1	2	3	4	5
Arm Position Matching	Absolute Error X	**0.870**	0.210	− 0.040		
	Absolute Error Y	0.320	**0.400**	− 0.230		
	Absolute Error XY	**0.910**	0.250	−0.100		
	Variability X	0.260	**0.810**	−0.130		
	Variability Y	0.140	**0.750**	−0.060		
	Variability XY	0.270	**0.860**	−0.140		
	Contraction Expansion Ratio X	−0.120	−0.040	**0.890**		
	Contraction Expansion Ratio Y	0.020	− 0.140	**0.800**		
	Contraction Expansion Ratio XY	−0.060	− 0.050	**0.990**		
	Shift X	**−0.550**	**0.580**	−0.070		
	Shift Y	0.250	**−0.580**	−0.070		
	Shift XY	**0.870**	−0.030	− 0.040		
Ball on Bar	Targets completed	**0.997**	−0.146	0.001		
	Mean movement time	**−0.965**	0.066	−0.037		
	Mean ball speed	**0.942**	0.083	0.029		
	Mean right hand speed	**0.913**	0.164	−0.017		
	Mean left hand speed	**0.956**	0.045	0.020		
	Right hand speed maxima	0.054	−0.012	**0.950**		
	Left hand speed maxima	0.175	−0.069	**0.766**		
	Mean bar angle rad	−0.067	**0.633**	−0.045		
	Stdev bar angle rad	−0.056	**0.801**	−0.201		
	Bar length variability*	0.250	0.335	0.145		
	Hand speed diff	**0.473**	**0.639**	0.108		
	Norm absolute hand speed diff	0.027	**0.849**	0.166		
	Hand speed peak bias	−0.263	0.113	**0.732**		
	Hand path bias*	−0.098	0.342	−0.191		
Visually Guided Reaching	Posture speed	−0.077	**0.519**	**0.512**		
	Reaction time	0.078	−0.162	**0.863**		
	Initial direction error	**0.547**	0.291	**0.435**		
	Initial distance ratio	**−0.915**	0.073	− 0.096		
	Speed maxima count	**0.780**	−0.324	−0.323		
	Min max speed difference	**0.738**	**0.568**	0.098		
	Movement time	0.105	**−0.950**	− 0.035		
	Path length ratio	**0.773**	**0.485**	0.142		
	Max speed	0.371	**0.847**	−0.148		
Reverse Visually Guided Reaching	Posture speed	−0.050	**0.614**	0.394		
	Reaction time	0.022	−0.016	**0.800**		
	Initial direction error	**0.818**	0.224	0.055		
	Initial distance ratio	**−0.856**	0.133	− 0.343		
	Initial speed ratio	**−0.806**	− 0.023	0.316		
	Speed maxima count	**0.837**	−0.101	0.302		
	Min max speed difference	0.263	**0.721**	0.053		
	Movement time	**0.621**	**−0.680**	0.229		
	Path length ratio	**0.547**	**0.692**	−0.013		
	Max speed	−0.248	**0.871**	−0.303		
	Correction time	**0.788**	0.095	−0.330		
Object Hit	Total hits	−0.028	0.137	**0.932**		
	Hits with left	**−0.612**	0.053	**0.658**		
	Hits with right	**0.545**	0.218	**0.758**		
	Median error	−0.046	0.119	**0.647**		
	Miss bias^a^	−0.040	− 0.022	− 0.365		
	Right hand speed	0.322	**0.847**	0.204		
	Movement area (right)	0.200	**0.881**	0.052		
	Left hand speed	−0.148	**0.888**	0.132		
	Movement area (left)	−0.219	**0.915**	0.061		
	Hand bias of hits	**0.893**	0.166	0.082		
	Hand transition	**−0.863**	− 0.008	0.051		
	Hand selection overlap	−0.056	**0.612**	0.094		
	Hand speed bias	**0.876**	0.037	0.140		
	Movement area bias	**0.719**	−0.290	−0.063		
Object Hit and Avoid	Total hits	−0.125	**0.933**	0.037	0.067	0.006
	Hits with left	−0.092	**0.780**	**−0.453**	0.034	0.233
	Hits with right	−0.061	**0.748**	**0.483**	0.070	−0.210
	Total Distractor hits	**0.982**	0.009	0.013	0.036	−0.012
	Distractor hits (left)	**0.925**	0.034	−0.020	−0.025	0.019
	Distractor hits (right)	**0.896**	−0.001	0.033	0.070	−0.027
	Median error	**−0.414**	**0.419**	0.076	0.073	−0.012
	Miss bias	−0.043	0.034	0.063	0.008	**0.914**
	Right hand speed	0.133	0.239	0.304	**0.724**	−0.025
	Movement area (right)	0.004	−0.054	0.360	**0.854**	0.050
	Left hand speed	0.133	0.215	−0.241	**0.764**	0.136
	Movement area (left)	−0.010	−0.035	− 0.327	**0.922**	− 0.125
	Hand bias of hits	−0.010	0.042	**0.809**	0.058	−0.387
	Hand transition	−0.060	0.030	**−0.824**	−0.082	− 0.390
	Hand selection overlap	0.021	−0.291	0.107	**0.568**	0.124
	Hand speed bias	0.003	0.045	**0.840**	0.007	−0.238
	Movement area bias	0.029	−0.029	**0.860**	−0.131	0.239
	Objects hit	**0.484**	**0.856**	0.003	0.037	−0.041
	Distractor proportion	**0.963**	−0.112	−0.003	0.027	−0.005
	Object processing rate	−0.380	**0.799**	0.000	0.071	0.041

Bold text indicates that the component loading is substantial (≥ .40). ^a^denotes that the KINARM parameter did no substantially load onto any component

### Arm position matching

Scree plot and parallel analysis yielded three models (1-, 2-, and 3-component solutions) that warranted further examination. For the 2- and 3-component solutions, the use of an orthogonal rotation was justified, as the inter-component correlations ranged from |-0.18| to |0.16|. Both the 1- and 2-component solutions included parameters that did not load onto any of the components (several loadings <|0.40|) or had sizeable cross loadings. Although several parameters had substantial loadings (≥|0.40|) on at least one component in the 3-component solution, the 3-component solution was the most interpretable (see Table 4 for the component loadings). The 3 components were related to 1) *position accuracy* (4, 25%), 2) *movement variability (6, 24%),* and 3) *contraction/expansion* (3, 21%).

### Ball on bar

Three models (1-, 2-, and 3-component solutions) were further examined based on the findings of the scree plot and parallel analysis. For the 2- and 3- component solutions, we used an oblique rotation because inter-component correlations ranged from |0.15| to |0.46|. Most parameters had substantial loadings (≥|0.40|) on at least one component in the 3-component solution. Bar length variability and hand path bias did not load substantially onto any of the 3 components. However, the 3-component solution was the most interpretable (see Table 4 for the component loadings). The three components were associated with 1) *speed and success* (6, 37%), 2) *bar angle* (4, 19%) and 3) hand-speed maxima which is related to *movement smoothness* (3, 16%). The contribution of *speed and success* to task performance suggests that speed is highly influential for high success rates.

### Visually guided reaching

Scree plot and parallel analysis yielded three models (1-, 2-, and 3-component solutions) for examination. For the 2- and 3-component solutions, we used an orthogonal rotation (varimax) as inter-component correlations ranged from |0.06| to |0.16|. Both 1- and 2-component solutions included a number of parameters that did not load onto any of the components (loadings <|0.40|) or had sizeable cross loadings, but all parameters had substantial loadings (≥|0.40|) on at least 1 component in the 3-component solution. The 3-component solution also was the most interpretable (see Table 4 for the component loadings). The components were related to: 1) *initial movement and smoothness* (5, 34%), 2) *total movement* (5, 30%), and 3) *posture and reaction time* (3, 15%).

### Reverse visually guided reaching

Scree plot and parallel analysis yielded three models (1-, 2-, and 3-component solutions) for further examination. For the 2- and 3-component solutions, we used an orthogonal rotation, as the inter-component correlations ranged from |-0.09| to |0.13|. Both the 1- and 2-component solutions included items that did not load onto any of the components (all loadings < |0.40|), but all items had substantial loadings (≥ |0.40|) on at least one component in the 3-component solution. The 3-component solution also was the most interpretable (see Table 4 for the component loadings). The 3 components were associated with: 1) *initial movement and smoothness* (7, 38%), 2) *total movement* (5, 24%), and 3) *reaction time* (1, 12%). *Initial movement and smoothness* accounted for more variance in this task than it did in VGR. Therefore, *initial movement* is imperative for success in RVGR.

### Object hit

According to the scree plot and parallel analysis, three models (1-, 2-, and 3-component solutions) warranted further examination. For the 2- and 3-component solutions, we used an orthogonal rotation, as the inter-component correlations ranged from |0.03| to |0.24|. Both the 1- and 2-component solutions included items that did not load onto any of the components (loadings <|0.40|) or had sizeable cross loadings, but all items had substantial loadings (≥|0.40|) on at least one component in the 3-component solution. The 3-component solution also was the most interpretable (see Table 4 for the component loadings). The 3 components were comprised of: 1) *hand bias* (6, 27%), 2) *speed and area* (5, 26%), and 3) *accuracy* (4, 18%). Hits with left and right hands cross loaded onto both *accuracy* and *hand bias*.

### Object hit and avoid

According to the scree plot and parallel analysis, five models (1-, 2-, 3-, 4- and 5-component solutions) warranted further examination. For the 2-, 3-, 4-, and 5-component solutions, we kept the oblique rotation because the inter-component correlations ranged from |-0.12| to |0.30|. The 1-, 2-, 3-, and 4-component solutions included items that did not load onto any of the components (all loadings < |0.40|) or had substantial cross loadings, but all items had substantial loadings (> |0.40|) on at least one component in the 5-component solution, which was also the most interpretable (see Table 4 for the component loadings). The 5 components were related to: 1) *impulsivity* (6, 21%), 2) *accuracy* (6, 20%), 3) *hand bias* (6, 18%)*,* 4) *speed and area* (5, 16%), and 5) *miss bias* (1, 7%). Three of these components (*hand bias, speed and area,* and *accuracy*) are shared with OH, whereas *miss bias* and *distractor avoidance* are components unique to OHA.

## Discussion

The KINARM robot produces granular datasets of performance metrics associated with sensory, motor, visuospatial, and executive function. The primary objective of this study was to reduce multivariate data produced by the KINARM and generate interpretable and intuitively understandable components across 6 behavioral tasks to improve characterization of participant performance. KINARM data dimensionality was substantially reduced, while still retaining a large proportion of variance. Therefore, complex participant data produced by the KINARM robot can be reduced into a small number of components that characterize participant performance.

### Interpretability and classification of components

We initially used PCA to reduce our multivariate dataset, and we then investigated if this statistical technique identified interpretable and classifiable components of participant performance. Within a task, components explained intuitive performance metrics. For example, in APM (a task that assesses limb proprioception), the 3 components identified were distinct measures of function (*position accuracy, movement variability* and *contraction/expansion)*. Furthermore, KINARM parameters were separated reproducibly across multiple tasks. For example, OHA was divided into 5 components: *impulsivity*, *accuracy*, *hand bias, speed and area*, and *miss bias.* Three of these components (*accuracy, hand bias,* and *speed and area*) are comparable to the PCA results observed from the OH task, thereby providing evidence that these tasks assess similar underlying behaviors. However, despite assessing similar underlying behaviors, the variance explained by each component differs. For example, *speed and area* accounts for more variability than *accuracy*, whereas in OHA *accuracy* accounts for more variability than *speed and area*. The addition of two unique components (i.e., *miss bias* and *impulsivity*) during the OHA may quantitatively represent the increase in cognitive load during this task. These two components that are absent from OH, and which account for 28% of overall variability, likely reflect the different impacts of impulsive movements in OH and OHA. In OH, quick and effectively impulsive movements are rewarded; quick movements with little inhibition will result in many targets being hit. In contrast, in OHA there is a penalty for impulsive movements that are not processed thoroughly prior to execution (i.e., deciding whether or not an object is a target or distractor prior to executing a movement towards it). Furthermore, behaviorally during the OH task, the subject can hit a total of 300 targets during the trial. In contrast, there are only 200 targets during OHA, plus 100 distractors. Therefore, *hand bias* and *speed and area* account for less variability in OHA, as now the subjects must inhibit their automatic responses (i.e., *impulsivity*), which also results in an increased need for improved *accuracy*. This contrast in cognitive load between tasks may anecdotally account for the difference in variance explained by the components mentioned above across OH and OHA. This result of similar inter-task grouping of parameters across related KINARM tasks was also observed during VGR and RVGR. Taken together, our results demonstrate the potential utility of PCA when applied to granular datasets, as well as the increased interpretability of KINARM parameters when categorized into behaviorally meaningful components that characterize performance.

Furthermore, parameter associations, positive or negative, strong or weak, within their respective components were logically related to participant performance. For example, the PCA of the BonB task identified the component *speed and success*. Targets completed, mean ball speed, mean right- and left-hand speed, and hand speed difference all had substantial positive loadings, whereas mean movement time had a substantial negative loading. Therefore, as participants increased their right- and left-hand speed, and thus ball speed, participants decrease the time from a target being displayed on screen to the target being reached by the participant, which results in a greater number of targets being completed throughout the task. In addition, the parameter norm absolute hand speed difference loaded substantially on *bar angle* but had a very low loading on *speed and area*. Behaviorally, this result is intuitive. If a participant reacts to a target quickly, the bar angle may become skewed if not carefully balanced, which is reflected by the substantial positive loadings of the other angle related parameters onto this component. These direction-based associations, low or high, were observed across most KINARM tasks and their respective parameters. These components will need to be further validated across KINARM platforms and patient populations. Overall, our analysis produced a consistent inter-task classification of behavioural variables (e.g., VGR and RVGR, OH and OHA), which demonstrates that these tasks assess similar underlying behaviours and further supports the validity of using this statistical technique on KINARM performance metrics. Our current analysis has substantially reduced these granular datasets into biologically plausible, interpretable, and behaviorally meaningful components.

### Cross-loading of parameters with multiple components

PCA indicated that some of the KINARM parameters cross-loaded with multiple components. For example, the APM task identified 3 components (*position accuracy, movement variability,* and *contraction/expansion),* which is fairly consistent with previous research that described these 3 variables as observed patterns of impairment after stroke [2]. Despite mostly a strong separation of these three components, shift x loaded onto both the *position accuracy* and *movement variability* components, with an inverse sign from negative to positive respectively. Shift indicates a systematic bias to move either left/right or front/back in the workspace. Therefore, in the *position accuracy* component, shift X (left/right) indicates, with a negative association, that shift X biases to the left as absolute error in the X and XY plane increases. In contrast, component 2, *movement variability*, indicates that as X shifts in the positive direction (i.e., hand moves to the right), overall variability increases.

Furthermore, certain parameters did not intuitively cross-load onto multiple components. For example, the OH task identified 3 components (*hand bias, speed and area,* and *accuracy*). Unexpectedly, the parameter total hits, which should be intuitively related to hits with left and right, did not cross-load onto the *accuracy* and *hand bias* components. However, this result demonstrates that total hits is highly related to *accuracy*, which is separable from *hand bias*. These components are not highly interrelated, as indicated by the use of orthogonal rotation, and measure separable aspects of participant performance. Therefore, *hand bias* measures the bias towards the participants limb preference, and use in space, which is separable from overall *accuracy* and the total targets being hit (e.g., right-handed participants may have biased use of their right hand, but this is separable from the number of targets that were accurately hit using either hand).

Interestingly, only three parameters did not substantially load onto any component: miss bias of OH and bar length variability and hand path bias from the BonB task. The reason for these low loadings is unclear. However, miss bias loaded highly onto a separate component for the OHA task, which may indicate that any bias of misses toward one side of the work space, or the other, is related to the increase in cognitive load during OHA. Furthermore, the low loadings for bar length variability and hand path bias may be the consequence of only analyzing level one of BonB, as this task increases with difficulty in the subsequent levels [19]. However, previous research has identified that level one of BonB identified the most performance impairments (i.e., highest number of parameters failed) in stroke participants, relative to control subjects [19]. Furthermore, the number of parameters failed tended to decrease with increasing task difficulty in successive levels, potentially due to the increased variability observed among controls, which ultimately influenced cut-off criteria used to quantify impairment among stroke participants [19]. Therefore, level one of BonB may be the most sensitive level used to detect impairment and is the most relevant for the current analysis. Future analysis may need to apply PCA to each level separately to investigate if the components are similar across all three levels. However, despite identifying some parameters that cross-load with multiple components and only a couple that did not substantially load onto any component, the majority of KINARM performance metrics did not cross-load with one another. Most components demonstrated high loadings with their respective components, which indicates a strong separation of components that quantify participant performance.

### Limitations and future directions

We excluded left-hand dominant participants, as many participants were right-handed, which means our findings may not generalize to left-handed participants. Furthermore, we could not conduct PCA across all task parameters, as participants did not complete all KINARM tasks. Therefore, we were unable to conduct PCA on broadly pooled performance scores across tasks, which confined our results to descriptions of each task only. However, the PCA has yielded task specific separations of components to further characterize sensorimotor function among healthy subjects, which may serve as a normative data set for future clinical comparisons of subject performance. In addition, two to three participants per task, except for RVGR, were missing participant data for education obtained. However, these missing data should not substantially impair our range of education. In addition, some tasks do not have Z-scores for all parameters being recorded (these metrics could not be standardized), and thus these parameters were excluded from this analysis. We conducted PCA only on the End-Point robot data, and therefore, we will need to conduct PCA on healthy participant data generated using the KINARM Exoskeleton. It is unclear how antigravity support provided by the Exoskeleton will affect participant performance and the generated components. Therefore, PCA of Exoskeleton data will be imperative to future investigations to implement PCA across KINARM platforms. Also, the current analysis was conducted in healthy participants, and future PCA will need to examine participant performance using a clinical sample, such as stroke, to validate these components and characterize performance in clinical populations to further demonstrate the utility of our current findings. Furthermore, the current analysis does not address individual or group differences in our healthy participant sample, which may complicate future comparisons between healthy subjects and clinical populations. However, using PCA across 6 KINARM tasks, which assesses a broad range of upper limb sensorimotor function, has led to a substantial reduction in the dimensionality of our data, and produced interpretable components of performance.

Therefore, PCA of KINARM data has the potential to become a valuable clinical tool. Applying PCA and identifying main sources of variability in a clinical examination can help make data from research tools, such as the KINARM, more concise and easily interpretable. Furthermore, identifying sources of strong variability may improve the detection of fluctuations in performance, which could increase the clinical relevance of robotic assessment as an evaluation tool. Therefore, analyzing data produced by a clinical population offers the potential to increase the clinical utility of the KINARM robot by maximizing interpretability of participant performance, while also minimizing information loss, to increase the characterization of performance among various populations. It is not clear if patient performance will result in similar components of performance patterns as healthy participants. Therefore, future applications of this analysis may offer potential insight into specific patterns of sensorimotor impairment among patient populations.

## Conclusions

Using PCA, granular KINARM performance data can be substantially reduced to a small number of interpretable components with minimal information loss and still accounting for a large amount of variance. To validate our current findings and to further characterize sensorimotor function and impairment, the components derived from our sample of healthy participants will serve as normative data for future comparisons to patient populations.

## Additional files

Additional file 1: Volunteer screening checklist. This form was administered to participants by trained researchers to screen for previous neurological and musculoskeletal complications. (PDF 53 kb)

Additional file 2: KINARM Standard Tests Parameter Tables. These tables provide detailed descriptions of each parameter per KINARM task. These tables were extracted from Dexterit-E 3.6 User Guide with permission from BKIN Technologies Ltd. (PDF 145 kb)

Additional file 3: Correlation matrices of KINARM parameters across all tasks. (DOCX 30 kb)

## Abbreviations

APM: Arm position matching
BonB: Ball on bar
KINARM: Kinesiological instrument for normal and altered reaching movements
OH: Object Hit
OH&A: Object hit and avoid
PCA: Principal component analysis
RVGR: Reverse visually guided reaching
VGR: Visually guided reaching
