# Supplementary material for: Using principal component analysis to reduce complex datasets produced by robotic technology in healthy participants
Source: J Neuroeng Rehabil. 2018 Jul 31;15:71. doi: 10.1186/s12984-018-0416-5 (PMC6069537; doi:10.1186/s12984-018-0416-5)
Supplement: Supplementary file 1 — Volunteer screening checklist. This form was administered to participants by trained researchers to screen for previous neurological and musculoskeletal complications. (PDF 53 kb) [file 12984_2018_416_MOESM1_ESM.pdf]

Volunteer Contact Information:

Name: ________________________________

Phone: _______________________ or Email: __________________________

What is your age? _________

Male / Female (Circle One or Highlight)

Neurological

Y N

☐ ☐ Have you ever had a stroke, mini-stroke or Transient Ischemic Attack (TIA)?

☐ ☐ Have you ever been diagnosed with a disease or condition affecting the brain or spinal cord (eg. Multiple Sclerosis, Parkinson's disease, Huntington's disease, Brain Aneurysm, Brain Tumor, Epilepsy)?

☐ ☐ Have you ever had a brain injury or closed head injury?

☐ ☐ Have you ever suffered a spinal cord injury?

☐ ☐ Have you ever had brain surgery?

☐ ☐ Have you ever had a radiculopathy or nerve root problem in the neck (cervical spine)?

☐ ☐ Have you ever had a brachial plexus injury (injury to a nerve in the shoulder(s)/arm(s))?

☐ ☐ Have you ever had any nerve injury/damage in your legs? (i.e. nerve impingement, sciatica?)

☐ ☐ Have you ever had damage to a nerve in the arms (peripheral nerve injury) (Carpal Tunnel Syndrome would be allowable)?

☐ ☐ Have you ever been diagnosed with neuropathy/peripheral neuropathy/diabetic neuropathy?

☐ ☐ Do you have numbness or tingling in your feet?

☐ ☐ Have you ever fainted?

Musculoskeletal

Y N

☐ ☐ Have you ever had a fracture to any of the bones of the shoulder, arm or wrist -including the collarbone (clavicle) or shoulder blade (scapula), the upper arm (humerus), the forearm (radius or ulna), the wrist (carpal bones)?

Side (L/R/Both) and bone(s)____________________________

☐ ☐ Do you have any injuries to the hips/legs or feet?

Are you receiving therapy or medication for these injuries? _______________________

☐ ☐ Have you had a joint replacement surgery (hip, knee or ankle)?

What joint(s) are affected on what side? ______________________________

☐ ☐ Do you have arthritis? (Osteo or Rheumatoid or other - circle)

What joint(s) are affected on what side? ______________________________

☐ ☐ Have you ever dislocated your shoulder? **Date and Side (L/R/Both)** ______________________

☐ ☐ Have you ever had a rotator cuff tear? **Date and Side (L/R/Both)**__________________________

☐ ☐ Have you received therapy (eg. physical therapy, massage, chiropractic) for a shoulder problem in the last 3-4 months? **(Y/N)** If Yes - do you still have problem with the shoulder?

Side(L/ R)___________________________

☐ ☐ Have you ever been diagnosed with a frozen shoulder (adhesive capsulitis)?

Date and Side (L/R/Both)____________________________

☐ ☐ Have you ever received a corticosteroid injection for a shoulder problem?

Date and Side (L/R/Both)______________________________________

☐ ☐ Have you ever had surgery to your shoulders, arms, forearms, wrists or hands? If Yes, Where was the surgery (eg. wrist) and what was the purpose ofthe surgery (eg. Carpal Tunnel release)?

☐ ☐ Do you currently have tennis elbow/golfer's elbow (medial or lateral epicondylitis?

Side (LIR/Both)____

☐ ☐ Do you have fibromyalgia or chronic pain affecting you neck, upper back or arms?

Side (L/R/Both)____

☐ ☐ Do you have a history of low back pain? ____

☐ ☐ Do you have a history of dizziness or imbalance (including vertigo, Meniere’s Disease, Vestibular disorder, medication that causes dizziness? ____

☐ ☐ Have you experienced a fall? (Unexpected loss of balance resulting in coming to rest on the floor, the ground or an object below knee level?

a. **If yes, how many times have you fallen?**____________________
b. **What were the circumstances that led to the fall?**_______________________________________

☐ ☐ Are you able to stand for about an hour at a time?
