# Supplementary material for: Using principal component analysis to reduce complex datasets produced by robotic technology in healthy participants
Source: J Neuroeng Rehabil. 2018 Jul 31;15:71. doi: 10.1186/s12984-018-0416-5 (PMC6069537; doi:10.1186/s12984-018-0416-5)
Supplement: Supplementary file 2 — KINARM Standard Tests Parameter Tables. These tables provide detailed descriptions of each parameter per KINARM task. These tables were extracted from Dexterit-E 3.6 User Guide with permission from BKIN Technologies Ltd. (PDF 145 kb) [file 12984_2018_416_MOESM2_ESM.pdf]

Arm Position Matching Parameters

Parameter	Meaning
Absolute Error X	The mean absolute distance error in the X direction across all trials.
Absolute Error Y	The mean absolute distance error in the Y direction across all trials.
Absolute Error XY	The mean absolute distance error across all trials.
Variability X	Mean value of the standard deviations of the subject's hand position in the X direction.
Variability Y	Mean value of the standard deviations of the subject's hand position in the Y direction.
Variability XY	Root-mean-square (RMS) of X and Y variabilities $var_{XY} = \sqrt{var_X^2 + var_Y^2}$
Contraction/ Expansion Ratio X	Ratio of range of movement in x-direction (arm moved by the subject compared to the arm moved by the robot). Range is calculated as the absolute difference between the mean positions of the left targets versus the right targets (i.e. for the 9 target protocol, the mean positions for the 3 left-most targets, relative to the subject, versus the mean positions for the 3 right-most targets). $ContExp_X = \frac{range_{X, arm\_moved\_by\_subject}}{range_{X, arm\_moved\_by\_robot}}$
Contraction/ Expansion Ratio Y	Ratio of range of movement in y-direction (arm moved by the subject compared to the arm moved by the robot). Range is calculated as the absolute difference between the mean positions of the distal targets versus the proximal targets (i.e. for the 9 target protocol, the mean positions for the 3 distal targets, relative to the subject in the y-direction, versus the mean positions for the 3 proximal targets).
Contraction/ Expansion Ratio XY	Ratio of the range of area moved over (arm moved by the subject compared to the arm moved by the robot). The area of the square made by the arm moved by the subject divided by the area of the square made by the arm moved by the robot.
Shift X	Mean difference between the mirrored X-position of the arm moved by the subject and the X-position of the arm moved by the robot. Positive values are for lateral shifts, negative values for medial shifts.
Shift Y	Mean difference between the mirrored Y-position of the arm moved by the subject and the Y-position of the arm moved by the robot. Positive values are for distal shifts, negative values for proximal shifts.
Shift XY	Root-mean-square (RMS) of the X and Y shifts. $shift_{XY} = shift_X^2 + shift_Y^2$

Ball on Bar Parameters

Type	Parameter	Meaning
Total Metric (TM)	Targets complete	The number of successful targets touched by the ball and held without a ball drop.
	Drops	The number of times the ball fell off the bar. The ball is fixed to the bar for level one so this parameter does not apply to that level.
	Drops / Targets	The ratio of Drops to Targets complete. [1]
Hand and Ball (HB)	Time to target	Mean time from a target being displayed to the first time that target is touched by the ball. On the first level the first 4 targets are not counted in this metric.[1]
	Ball speed	The mean speed of the ball during the level.[2]
	Right hand speed	Mean speed of the right hand during the level.[2]
	Left hand speed	Mean speed of the left hand during the level.[2]
	Right hand speed peaks	Count of the speed maxima for the right hand for the level.[2]
	Left hand speed peaks	Count of the speed maxima for the left hand for the level.[2]
Bi-Manual (BM)	Mean bar tilt	Mean angle of the bar during the level.[2]
	Bar tilt stdev	Standard deviation of the bar angle during the level.[2]
	Bar length variability	Calculated as the standard deviation of bar length / mean bar length for the level. This is also known as the coefficient of variation. [2]
Inter-Limb (IL)	Reaction time: abs diff	Calculated as the mean of abs(RH reaction time - LH reaction time) for each completed target in a level. The reaction time for levels 2 and 3 are not calculated because the movement of the ball (and therefore the hands to stabilize) makes an exact reaction time difficult to interpret. Valid reaction times for both hands must be found for at least 5 completed targets, otherwise this value is reported as NaN. The first four successful reaches are excluded from this calculation.
	Hand speed diff	Calculated as sum(abs(RHS-LHS)) / (sum(RHS + LHS) / 2) where RHS = Right hand speed and LHS = Left hand speed, where each sum is the sum over all kinematic frames. [2]
	Hand speed peaks bias	Calculated as (DomHSP - NDomHSP) / (DomHSP + NDomHSP) where DomHSP = Dominant hand speed peaks and NDomHSP = Non dominant hand speed peaks. [2]

	Hand path length bias	This is calculated as (DomHPL - NDomHPL) / (DomHPL + NDomHPL) where DomHPL = Dominant hand path length and NDomHPL = Non dominant hand path length. [2]

[1] If no targets are complete for the level, this parameter is reported as NaN (Not a number).

[2] Data from after the ball leaves the bar until the next target is touched is not included in the calculation because the ball is not behaving as it would normally be during the level, i.e. it is fixed to the center of the bar.

Visually Guided Reaching Parameters

Type	Parameter Name	Meaning
NT - No trial	No init stabilization	Count of trials where the subject failed to stabilize at the starting target. Inter-limb comparisons are not performed on this statistic because the difference is typically 0 for healthy control subjects.
	No reaction time	Count of trials where no movement onset could be calculated for any reason. Inter-limb comparisons are not performed on this statistic because the difference is typically 0 for healthy control subjects.
	No end movement	The number of trials for which the destination target was not reached[2] or it was reached but end of movement is not detected algorithmically (see movement offset[4] definition). Inter-limb comparisons are not performed on this statistic because the difference is typically 0 for healthy control subjects.
	End target not reached	Count of trials where the end target was not stabilized in for any reason. Inter-limb comparisons are not performed on this statistic because the difference is typically 0 for healthy control subjects.
PC - Posture Control	Posture Speed	The median hand speed when the hand should be at rest. The median value of all trials is reported.
VR - Visual Reaction	Reaction Time	The time between illumination of the destination target and movement onset[1]. The median value of all trials[2] is reported. Note: the time from a target display command until it is actually displayed on the screen is approximately 50ms, this extra 50ms is included in the Reaction Time.
FM - First Movement	Initial Direction Angle	The angular deviation between (a) a straight line from the hand position at movement onset[1] to the hand position after the initial phase of movement[3] and (b) a straight line from the hand position at movement onset to the destination target. For each trial, the absolute value of this deviation is calculated and used. The median value of all trials[2] is reported.
	Initial Distance Ratio	The ratio of (a) the distance the hand travelled during the subject's initial phase of movement[3] to (b) the distance the hand travelled between movement onset[1] and movement offset[4]. The median value of all trials[2] is reported.

	Initial Speed Ratio	The ratio of (a) the maximum hand speed during the subject's initial phase of movement[3] to (b) the maximum hand speed between movement onset[1] and movement offset[4] (i.e. Max Speed). The mean value of all trials[2] is reported.
CM - Corrective Movement	Speed Maxima Count	The number of maxima in hand speed between movement onset[1] and movement offset[4]. The mean value of all trials[2] is reported.
	Min-Max Speed	The mean difference between pairs of adjacent local hand speed minima and maxima, for all such pairs between the time of Max Speed, and movement offset[4]. The mean value of all trials[2] is reported.
TM - Total Movement	Movement time	The total time elapsed from movement onset[1] to movement offset[4]. The median value of all trials[2] is reported.
	Path length ratio	The ratio of (a) the distance travelled by the hand between movement onset[1] and movement offset[4]and (b) the straight line distance between those two hand positions. The mean value of all trials[2] is reported.
	Max Speed	The maximum hand speed between movement onset[1] and movement offset[4]. The median value of all trials[2] is reported.

[1] *Movement onset is nominally the time that movement towards the destination target was detected, if such a movement can be detected prior to a time-out event. If such a movement cannot be detected algorithmically then movement onset is set to the first time that the subject left the initial target following illumination of the destination target. Movement onset is not defined if the hand never reaches the initial target, never stabilizes at the initial target or never leaves the initial target.*

[2] *Excluding trials for which movement onset was not defined or a false start was detected. A false start is defined by too fast a reaction time to be a true reaction.*

[3] *Initial phase of movement is used here to mean the time from movement onset to the earlier of the first speed minima after movement onset or movement offset[4].*

[4] *Movement offset is nominally the time at which the subject finished their movement in the destination target. If the destination target was not reached or if it was reached but end of movement was not detected algorithmically then No Movement End count is increased and movement offset is set to the end of the trial, which is the next time a visual cues changes (i.e. when the destination target turns off for 8 target reach protocols or when the next trial's destination target turns on for 4 target in and out protocols). Movement offset is always defined if movement onset is defined.*

NOTE: Inter-limb values are always calculated as (Dominant arm value) - (Non-dominant arm value).

Visually Guided Reaching Additional CSV parameters

Name	Meaning
False starts	The count of trials where the movement start was detected <130ms after the destination target was turned on.

Reverse Visually Guided Reaching parameters

Type	Parameter	Meaning
First Movement (FM)	Direction errors	The count of times the subject moved the hand feedback away from the destination target as part of the first movement.
	Correction time	The mean amount of time the subject took for each target to start moving the hand feedback towards the destination target. If the subject's initial movement was toward the destination target then the value for that trial is zero. Otherwise this is the amount of time from the destination target turning on to the time the subject was the farthest from the destination target.

Additional Parameters in CSV export

See **"Visually Guided Reaching"** for additional parameters in CSV export for Reverse Visually Guided Reaching.

Object Hit Parameters

Type	Parameter	Meaning
Task level	Target hits	A ball is considered to be 'hit' if its final trajectory moves it off the screen anywhere other than the bottom of the screen (i.e. other than the border nearest the subject). This parameter is reported as the percentage of total balls dropped.
	Median Error	The percentage of the way through the task when the subject made half of his or her errors.
	Miss Bias	Quantifies any bias of misses toward one side of the work space or the other (x direction only). This parameter is reported in cm to show where in the work space the bias is located. It is computed by counting the number of misses for each of the ten bins and then calculating the weighted mean of the resulting distribution over the ten bins, as defined below (x_i is the x location of the ith bin, and the weight w_i is the number of misses in the ith bin). . $\text{weighted\_mean} = \frac{\sum_{i=1}^{10} x_i \cdot w_i}{\sum_{i=1}^{10} w_i}$

Left/Right hand	Hand Speed	The mean hand speed the subject maintained through the entire task.
	Movement Area	The area of space the subject used with each hand during the task. This parameter is determined by defining a convex hull that encompasses the subject's complete hand path.
Inter-limb	Hand Bias Hits	Quantifies which hand is used more often for hitting the balls (hand dominance). It is calculated as (RHH – LHH) / (RHH + LHH).
	Hand transition	Shows where the subject's preference for using one hand over the other switches in the work space. It is computed by taking the mean of two values: the right hand and the left hand weighted means of hit distributions. The weighted mean of hit distributions for each hand are calculated independently for each hand using a subset of bins, including only those where both hands made hits (overlapping bins) plus one additional bin on each side of the overlap bins. In the special case where no overlap occurs, the subset of bins used includes the right most bin in which hits were made by the left hand and the left-most bin where hits were made with the right hand.
	Hand Selection Overlap	Captures how effective subjects are at using both hands and how often they overlap hands (i.e. hit balls with both the right and left hands in the same area of the work space). This parameter is reported as the summation of hand switches for each bin, divided by the total hits.
	Hand Speed Bias	A value from -1 to 1 which describes the bias in Hand Speed between the hands. It is calculated as (RHS - LHS) / (RHS + LHS)
	Movement Area Bias	A value from -1 to 1 which describes the bias in Movement Area between the hands. It is calculated as (RMA - LMA) / (RMA + LMA)

Additional Parameters in CSV export

Some parameters are provided in the CSV export, but are not available in the report. The parameters that are in the CSV export but not in the report are listed below.

Object Hit Additional CSV Parameters

Name	Meaning
Total hits	The count of objects successfully hit. This value is out of a possible 300.
Hits with Left	The count of objects successfully hit with the left hand.
Hits with Right	The count of objects successfully hit with the right hand.

Object Hit and Avoid Parameters

Parameter	Meaning
Distracter Hits	The count of distracter objects the subject hit. It is reported as the percentage of total distracters dropped.

The parameters collected for this task include those for the Object Hit task, plus the extra parameter outlined above (i.e., distractor hits).

Object Hit and Avoid Additional CSV Parameters

Name	Meaning
Total hits	The count of targets successfully hit. This value is out of a possible 200.
Hits with Left	The count of targets successfully hit with the left hand.
Hits with Right	The count of targets successfully hit with the right hand.
Distracter hits total	The count of distracter objects hit. This value is out of a possible 100.
Distracter hits left	The count of distracter objects hit with the left hand.
Distracter hits right	The count of distracter objects hit with the left hand.
