# Supplementary material for: Using principal component analysis to reduce complex datasets produced by robotic technology in healthy participants
Source: J Neuroeng Rehabil. 2018 Jul 31;15:71. doi: 10.1186/s12984-018-0416-5 (PMC6069537; doi:10.1186/s12984-018-0416-5)
Supplement: Supplementary file 3 — Correlation matrices of KINARM parameters across all tasks. (DOCX 30 kb) [file 12984_2018_416_MOESM3_ESM.docx]

**Table**. Correlation Matrices of KINARM Task Parameters.

**Arm Position Matching:**

| **Parameters** | **Absolute error X** | **Absolute error Y** | **Absolute error XY** | **Variability X** | **Variability Y** | **Variability XY** | **Contraction expansion ratio X** | **Contraction expansion ratio Y** | **Contraction expansion ratio XY** | **Shift X** | **Shift Y** |
| --- | --- | --- | --- | --- | --- | --- | --- | --- | --- | --- | --- |
| Absolute error X |  |  |  |  |  |  |  |  |  |  |  |
| Absolute error Y | 0.201** |  |  |  |  |  |  |  |  |  |  |
| Absolute error XY | 0.900*** | 0.418*** |  |  |  |  |  |  |  |  |  |
| Variability X | 0.416*** | 0.189* | 0.390*** |  |  |  |  |  |  |  |  |
| Variability Y | 0.175* | 0.530*** | 0.285*** | 0.508*** |  |  |  |  |  |  |  |
| Variability XY | 0.398*** | 0.267*** | 0.409*** | 0.983*** | 0.634*** |  |  |  |  |  |  |
| Contraction expansion ratio X | -0.163* | -0.141 | -0.200** | -0.224** | -0.062 | -0.238** |  |  |  |  |  |
| Contraction expansion ratio Y | -0.064 | -0.299*** | -0.122 | -0.158* | -0.154* | -0.169* | 0.479*** |  |  |  |  |
| Contraction expansion ratio XY | -0.114 | -0.198** | -0.158* | -0.186* | -0.096 | -0.202** | 0.929*** | 0.737*** |  |  |  |
| Shift X | -0.260*** | 0.122 | -0.254*** | 0.204** | 0.263*** | 0.228** | -0.014 | -0.152* | -0.057 |  |  |
| Shift Y | 0.010 | -0.084 | 0.009 | -0.259*** | -0.202** | -0.261*** | -0.041 | 0.062 | -0.023 | -0.508*** |  |
| Shift XY | 0.678*** | 0.345*** | 0.777*** | 0.117 | 0.079 | 0.124 | -0.114 | -0.028 | -0.091 | -0.376*** | 0.076 |

**Ball on Bar:**

| **Parameters** | **Targets completed** | **Mean movement time** | **Mean ball speed** | **Mean right hand speed** | **Mean left hand speed** | **Right hand speed maxima** | **Left hand speed maxima** | **Mean bar angle** | **Stdev bar angle** | **Bar length variability** | **Hand speed diff** | **Norm absolute hand speed diff** | **Hand speed peak bias** |
| --- | --- | --- | --- | --- | --- | --- | --- | --- | --- | --- | --- | --- | --- |
| Targets completed |  |  |  |  |  |  |  |  |  |  |  |  |  |
| Mean movement time | -0.941*** |  |  |  |  |  |  |  |  |  |  |  |  |
| Mean ball speed | 0.901*** | -0.924*** |  |  |  |  |  |  |  |  |  |  |  |
| Mean right hand speed | 0.858*** | -0.893*** | 0.985*** |  |  |  |  |  |  |  |  |  |  |
| Mean left hand speed | 0.905*** | -0.920*** | 0.987*** | 0.962*** |  |  |  |  |  |  |  |  |  |
| Right hand speed maxima | 0.469*** | -0.499*** | 0.496*** | 0.456*** | 0.475*** |  |  |  |  |  |  |  |  |
| Left hand speed maxima | 0.471*** | -0.488*** | 0.484*** | 0.448*** | 0.456*** | 0.926*** |  |  |  |  |  |  |  |
| Mean bar angle | 0.087 | -0.117 | 0.188** | 0.225** | 0.183** | 0.038 | 0.025 |  |  |  |  |  |  |
| Stdev bar angle | 0.084 | -0.117 | 0.175* | 0.200** | 0.168* | -0.070 | -0.080 | 0.329*** |  |  |  |  |  |
| Bar length variability | 0.306*** | -0.365*** | 0.429*** | 0.464*** | 0.404*** | 0.269*** | 0.256*** | 0.157* | 0.104 |  |  |  |  |
| Hand speed diff | 0.602*** | -0.658*** | 0.786*** | 0.809*** | 0.768*** | 0.409*** | 0.380*** | 0.338*** | 0.484*** | 0.474*** |  |  |  |
| Norm absolute hand speed diff | 0.259*** | -0.316*** | 0.448*** | 0.479*** | 0.431*** | 0.271*** | 0.238*** | 0.337*** | 0.572*** | 0.371*** | 0.898*** |  |  |
| Hand speed peak bias | 0.144* | -0.181** | 0.181** | 0.156* | 0.189** | 0.480*** | 0.119 | 0.039 | -0.016 | 0.111 | 0.186** | 0.153* |  |
| Hand path bias | -0.132 | 0.065 | -0.026 | 0.109 | -0.150* | -0.094 | -0.050 | 0.129 | 0.074 | 0.157* | 0.069 | 0.081 | -0.147* |

**Visually Guided Reaching:**

| **Parameters** | **Posture speed** | **Reaction time** | **Initial direction angle** | **Initial distance ratio** | **Speed maxima count** | **Min max speed difference** | **Movement time** | **Path length ratio** |
| --- | --- | --- | --- | --- | --- | --- | --- | --- |
| Posture speed |  |  |  |  |  |  |  |  |
| Reaction time | 0.179* |  |  |  |  |  |  |  |
| Initial direction angle | 0.201** | 0.221** |  |  |  |  |  |  |
| Initial distance ratio | -0.073 | -0.125 | -0.441*** |  |  |  |  |  |
| Speed maxima count | -0.270*** | -0.102 | 0.111 | -0.708*** |  |  |  |  |
| Min max speed difference | 0.251*** | 0.058 | 0.533*** | -0.614*** | 0.291*** |  |  |  |
| Movement time | -0.454*** | 0.045 | -0.213** | -0.149* | 0.344*** | -0.432*** |  |  |
| Path length ratio | 0.204** | 0.099 | 0.588*** | -0.614*** | 0.321*** | 0.918*** | -0.334*** |  |
| Max speed | 0.257*** | -0.114 | 0.349*** | -0.261*** | 0.122 | 0.686*** | -0.805*** | 0.622*** |

**Reverse Visually Guided Reaching:**

| **Parameters** | **Posture speed** | **Reaction time** | **Initial direction angle** | **Initial distance ratio** | **Initial speed ratio** | **Speed maxima count** | **Min max speed difference** | **Movement time** | **Path length ratio** | **Max speed** |
| --- | --- | --- | --- | --- | --- | --- | --- | --- | --- | --- |
| Posture speed |  |  |  |  |  |  |  |  |  |  |
| Reaction time | 0.157 |  |  |  |  |  |  |  |  |  |
| Initial direction angle | 0.049 | 0.022 |  |  |  |  |  |  |  |  |
| Initial distance ratio | -0.034 | -0.178 | -0.699*** |  |  |  |  |  |  |  |
| Initial speed ratio | -0.001 | 0.102 | -0.568*** | 0.539*** |  |  |  |  |  |  |
| Speed maxima count | 0.027 | 0.118 | 0.614*** | -0.855*** | -0.508*** |  |  |  |  |  |
| Min max speed difference | 0.241* | -0.069 | 0.408*** | -0.178 | -0.115 | 0.186 |  |  |  |  |
| Movement time | -0.395*** | 0.160 | 0.353*** | -0.666*** | -0.348*** | 0.674*** | -0.229* |  |  |  |
| Path length ratio | 0.257** | 0.060 | 0.540*** | -0.323*** | -0.365*** | 0.383*** | 0.586*** | -0.085 |  |  |
| Max speed | 0.359*** | -0.207* | -0.024 | 0.435*** | 0.093 | -0.347*** | 0.490*** | -0.817*** | 0.483*** |  |
| Correction time | -0.025 | -0.042 | 0.543*** | -0.471*** | -0.840*** | 0.452*** | 0.081 | 0.309** | 0.540*** | -0.010 |

**Object Hit:**

| **Parameters** | **Total hits** | **Hits with left** | **Hits with right** | **Median error** | **Miss bias** | **Hand speed right** | **Movement area right hand** | **Hand speed left** | **Movement area left hand** | **Hand bias of hits** | **Hand transition** | **Hand selection overlap** | **Hand speed bias** |
| --- | --- | --- | --- | --- | --- | --- | --- | --- | --- | --- | --- | --- | --- |
| Total.hits |  |  |  |  |  |  |  |  |  |  |  |  |  |
| Hits with left | 0.685*** |  |  |  |  |  |  |  |  |  |  |  |  |
| Hits with right | 0.741*** | 0.152* |  |  |  |  |  |  |  |  |  |  |  |
| Median error | 0.458*** | 0.365*** | 0.385*** |  |  |  |  |  |  |  |  |  |  |
| Miss bias | -0.234** | 0.004 | -0.316*** | -0.093 |  |  |  |  |  |  |  |  |  |
| Hand speed right | 0.315*** | 0.014 | 0.495*** | 0.167* | -0.067 |  |  |  |  |  |  |  |  |
| Movement area right hand | 0.174* | 0.014 | 0.299*** | 0.185* | 0.002 | 0.749*** |  |  |  |  |  |  |  |
| Hand speed left | 0.276*** | 0.227** | 0.216** | 0.147* | -0.051 | 0.850*** | 0.677*** |  |  |  |  |  |  |
| Movement area left hand | 0.170* | 0.180* | 0.139 | 0.209** | -0.081 | 0.644*** | 0.839*** | 0.762*** |  |  |  |  |  |
| Hand bias of hits | 0.032 | -0.614*** | 0.664*** | 0.025 | -0.232** | 0.404*** | 0.256*** | 0.013 | 0.004 |  |  |  |  |
| Hand transition | 0.047 | 0.477*** | -0.411*** | 0.018 | -0.158* | -0.250*** | -0.176* | 0.095 | 0.205** | -0.692*** |  |  |  |
| Hand selection overlap | 0.163* | 0.181* | 0.169* | 0.102 | -0.040 | 0.404*** | 0.471*** | 0.436*** | 0.484*** | 0.035 | 0.001 |  |  |
| Hand speed bias | 0.094 | -0.395*** | 0.552*** | 0.054 | -0.036 | 0.383*** | 0.220** | -0.153* | -0.130 | 0.749*** | -0.653*** | 0.003 |  |
| Movement area bias | -0.063 | -0.342*** | 0.192** | -0.117 | 0.150* | -0.004 | 0.037 | -0.334*** | -0.505*** | 0.397*** | -0.649*** | -0.162* | 0.575*** |

**Object Hit and Avoid:**

| **Parameters** | **Total hits** | **Hits with left** | **Hits with right** | **Distractor hits total** | **Distractor hits left** | **Distractor hits right** | **Median error** | **Miss bias** | **Hand speed right** | **Movement area right hand** | **Hand speed left** | **Movement area left hand** | **Hand bias of hits** | **Hand transition** | **Hand selection overlap** | **Hand speed bias** | **Movement area bias** | **Objects hit** | **Distractor proportion** |
| --- | --- | --- | --- | --- | --- | --- | --- | --- | --- | --- | --- | --- | --- | --- | --- | --- | --- | --- | --- |
| Total hits |  |  |  |  |  |  |  |  |  |  |  |  |  |  |  |  |  |  |  |
| Hits with left | 0.770*** |  |  |  |  |  |  |  |  |  |  |  |  |  |  |  |  |  |  |
| Hits with right | 0.812*** | 0.306*** |  |  |  |  |  |  |  |  |  |  |  |  |  |  |  |  |  |
| Distractor hits total | -0.203** | -0.221** | -0.060 |  |  |  |  |  |  |  |  |  |  |  |  |  |  |  |  |
| Distractor hits left | -0.199** | -0.190* | -0.078 | 0.919*** |  |  |  |  |  |  |  |  |  |  |  |  |  |  |  |
| Distractor hits right | -0.167* | -0.208** | -0.035 | 0.918*** | 0.700*** |  |  |  |  |  |  |  |  |  |  |  |  |  |  |
| Median error | 0.444*** | 0.352*** | 0.367*** | -0.351*** | -0.292*** | -0.353*** |  |  |  |  |  |  |  |  |  |  |  |  |  |
| Miss bias | 0.048 | 0.209** | -0.154* | -0.101 | -0.081 | -0.080 | 0.062 |  |  |  |  |  |  |  |  |  |  |  |  |
| Hand speed right | 0.425*** | 0.164* | 0.547*** | 0.334*** | 0.264*** | 0.341*** | 0.138 | -0.037 |  |  |  |  |  |  |  |  |  |  |  |
| Movement area right hand | 0.240** | 0.029 | 0.369*** | 0.283*** | 0.219** | 0.283*** | 0.072 | -0.024 | 0.789*** |  |  |  |  |  |  |  |  |  |  |
| Hand speed left | 0.375*** | 0.432*** | 0.225** | 0.286*** | 0.259*** | 0.257*** | 0.095 | 0.083 | 0.789*** | 0.608*** |  |  |  |  |  |  |  |  |  |
| Movement area left hand | 0.248** | 0.278*** | 0.130 | 0.237** | 0.181* | 0.253*** | 0.070 | -0.045 | 0.605*** | 0.683*** | 0.739*** |  |  |  |  |  |  |  |  |
| Hand bias of hits | 0.105 | -0.496*** | 0.626*** | 0.108 | 0.062 | 0.123 | 0.091 | -0.304*** | 0.384*** | 0.337*** | -0.138 | -0.087 |  |  |  |  |  |  |  |
| Hand transition | -0.022 | 0.299*** | -0.341*** | -0.131 | -0.106 | -0.133 | -0.012 | -0.230** | -0.317*** | -0.381*** | 0.010 | 0.132 | -0.533*** |  |  |  |  |  |  |
| Hand selection overlap | -0.013 | -0.096 | 0.039 | 0.214** | 0.154* | 0.229** | -0.116 | 0.084 | 0.252*** | 0.355*** | 0.195* | 0.328*** | 0.077 | -0.124 |  |  |  |  |  |
| Hand speed bias | 0.102 | -0.385*** | 0.515*** | 0.095 | 0.022 | 0.149 | 0.074 | -0.175* | 0.394*** | 0.338*** | -0.247** | -0.146 | 0.812*** | -0.512*** | 0.106 |  |  |  |  |
| Movement area bias | -0.031 | -0.321*** | 0.272*** | 0.053 | 0.047 | 0.032 | -0.019 | 0.042 | 0.197* | 0.349*** | -0.192* | -0.441*** | 0.517*** | -0.640*** | 0.022 | 0.600*** |  |  |  |
| Objects hit | 0.754*** | 0.562*** | 0.666*** | 0.385*** | 0.338*** | 0.387*** | 0.125 | -0.046 | 0.545*** | 0.333*** | 0.479*** | 0.342*** | 0.148 | -0.052 | 0.099 | 0.132 | -0.031 |  |  |
| Distractor proportion | -0.319*** | -0.310*** | -0.167* | 0.991*** | 0.912*** | 0.906*** | -0.396*** | -0.096 | 0.259*** | 0.239** | 0.227** | 0.200** | 0.075 | -0.117 | 0.215** | 0.066 | 0.047 | 0.265*** |  |
| Object processing rate | 0.867*** | 0.720*** | 0.677*** | -0.421*** | -0.376*** | -0.406*** | 0.552*** | 0.088 | 0.277*** | 0.139 | 0.288*** | 0.175* | 0.033 | 0.008 | -0.098 | 0.003 | -0.063 | 0.480*** | -0.511*** |

* p < .05. ** p < .01. *** p < .001.
